# Translating glucose tolerance data from mice to humans: Insights from stable isotope labelled glucose tolerance tests

**DOI:** 10.1016/j.molmet.2021.101281

**Published:** 2021-06-24

**Authors:** Clinton R. Bruce, Steven Hamley, Teddy Ang, Kirsten F. Howlett, Christopher S. Shaw, Greg M. Kowalski

**Affiliations:** 1 Institute for Physical Activity and Nutrition, School of Exercise and Nutrition Science, Deakin University, Geelong, Waurn Ponds, Victoria, 3216, Australia; 2 Metabolic Research Unit, School of Medicine, Deakin University, Geelong, Waurn Ponds, Victoria, 3216, Australia

**Keywords:** OGTT, Mouse, Human, Stable isotope, Endogenous glucose production

## Abstract

**Objective:**

The glucose tolerance test (GTT) is widely used in human and animal biomedical and pharmaceutical research. Despite its prevalent use, particularly in mouse metabolic phenotyping, to the best of our knowledge we are not aware of any studies that have attempted to qualitatively compare the metabolic events during a GTT in mice with those performed in humans.

**Methods:**

Stable isotope labelled oral glucose tolerance tests (siOGTTs; [6,6-^2^H_2_]glucose) were performed in both human and mouse cohorts to provide greater resolution into postprandial glucose kinetics. The siOGTT allows for the partitioning of circulating glucose into that derived from exogenous and endogenous sources. Young adults spanning the spectrum of normal glucose tolerance (n = 221), impaired fasting (n = 14), and impaired glucose tolerance (n = 19) underwent a 75g siOGTT, whereas a 50 mg siOGTT was performed on chow (n = 43) and high-fat high-sucrose fed C57Bl6 male mice (n = 46).

**Results:**

During the siOGTT in humans, there is a long period (>3hr) of glucose absorption and, accordingly, a large, sustained insulin response and robust suppression of lipolysis and endogenous glucose production (EGP), even in the presence of glucose intolerance. In contrast, mice appear to be highly reliant on glucose effectiveness to clear exogenous glucose and experience only modest, transient insulin responses with little, if any, suppression of EGP. In addition to the impaired stimulation of glucose uptake, mice with the worst glucose tolerance appear to have a paradoxical and persistent rise in EGP during the OGTT, likely related to handling stress.

**Conclusions:**

The metabolic response to the OGTT in mice and humans is highly divergent. The potential reasons for these differences and their impact on the interpretation of mouse glucose tolerance data and their translation to humans are discussed.

## Highlights

• We compared the mechanisms governing glucose handling in humans and mice.
• Humans and mice underwent stable isotope labelled oral glucose tolerance tests.
• Metabolic responses between humans and mice were highly divergent.
• Unlike humans, most mice exhibit little EGP suppression or insulin response.

## 1 Introduction

The glucose tolerance test (GTT) is a cornerstone technique that assesses whole-body glucose homeostasis following the administration of a glucose load. It is not only used in clinical practice to detect glucose intolerance (prediabetes) and diabetes but is also widely used in basic and clinical human metabolic studies and animal-based biomedical and pharmaceutical research. The homeostatic response to an ingested glucose bolus requires the coordinated regulation of numerous body systems that stimulate tissue glucose disposal, inhibit endogenous glucose production, and regulate intestinal glucose entry [1]. Accordingly, glucose handling during the GTT is dependent on numerous integrated factors, of which insulin secretion, insulin action, and glucose effectiveness (i.e., the ability of hyperglycemia to regulate glucose uptake and production under basal/constant insulin concentrations) are particularly important [[1], [2], [3]]. Given the high prevalence of the metabolic syndrome and type 2 diabetes (T2D), there is great scientific and medical interest in understanding the pathogenesis and treatment of disordered glucose metabolism. With the emergence of modern-day genetic engineering techniques, the laboratory mouse, particularly the male high-fat high-sucrose (HFHS) fed C57Bl/6 strain, has become a major mechanistic research tool used to emulate the pathogenesis of human metabolic disease, including glucose intolerance, insulin resistance, hyperinsulinemia, obesity, fatty liver, and cardiovascular disease [1,4,5]. In this context, the GTT (oral, intraperitoneal, or intravenous) is frequently used in laboratory mice as an integrative readout of whole-body glucose handling and, when combined with blood insulin measurements, is thought to provide some additional readout of β-cell function and insulin action [6].

It is noteworthy that numerous papers have discussed the issues, challenges, and guidelines that relate to the metabolic phenotyping of mice [[7], [8], [9], [10], [11], [12], [13]]. Key topics discussed include the effects of glucose dosing, fasting duration, handling stress, anaesthesia, and the timing of experimentation from the standpoint of assessing murine glucose metabolism, particularly regarding glucose tolerance testing [[7], [8], [9], [10], [11], [12],^14^]. Additionally, we have discussed the critical differences in glucose metabolism between humans and rodents, along with the potential considerations when attempting to translate rodent data to a human context [1]. However, despite the GTT being one of the most widely used tests in the field of mammalian glucose metabolism, to the best of our knowledge, we are not aware of any publications that have attempted to qualitatively compare postprandial glucose handling in humans with that of mice. Based on our experience of metabolically phenotyping hundreds of humans [[15], [16], [17], [18], [19]] and C57Bl/6 mice [[20], [21], [22], [23], [24], [25], [26], [27]], particularly using stable isotope labelled GTTs (siGTT), we noticed that there are important differences in glucose handling that do not appear to have been adequately addressed in the literature. Accordingly, we aimed to shed light on these observations and evaluate whether the metabolic events that occur during a GTT in mice resemble those that occur in humans.

Adding stable isotope labelled glucose (i.e., [6,6-^2^H_2_]glucose) to the glucose drink (or injected bolus) has the benefit of providing additional mechanistic and kinetic insight into postprandial glucose handling during the GTT [[28], [29], [30], [31]]. Specifically, via mass-spectrometry or NMR, it is possible to quantify the amount of glucose that has been derived from the exogenous (oral or systemically injected) glucose load as well as the amount of natural unlabelled glucose that originates from endogenous glucose production (EGP) via the liver and kidneys (i.e., endogenous glucose; [[28], [29], [30], [31], [32]]). The siGTT *does not allow absolute postprandial glucose flux rates* (i.e., mg.min^−1^) to be calculated on its own. However, it *does* permit the ability to quantify the accumulation and disappearance of the administered glucose load in the blood, thus providing insight into glucose production and disposal patterns. Furthermore, in humans, the oral siGTT (siOGTT) can be combined with mathematical models, such as the oral minimal model, to determine indices of insulin action on glucose disposal and EGP suppression [15,[33], [34], [35]]. If one wishes to perform more complex glucose tracer experiments in humans (i.e., dual and triple tracer experiments), it becomes possible to determine *absolute rates* of whole-body glucose disposal, meal glucose systemic appearance, and EGP. Here, the siOGTT or mixed meal tolerance tests can be combined with variable infusions of multiple stable isotopes (e.g., [1–^13^C], [1–^2^H], or [U–^13^C_6_]glucose) or radioactive glucose tracers in a manner that mimics the anticipated exogenous and endogenous glucose concentrations [[16], [17], [18], [19],^28^,^29^,[36], [37], [38]]. When combined with frequent blood sampling, these techniques have shown that glucose ingestion causes a rapid (within 30 min) and sustained, but not complete, suppression of EGP (55–80% of basal) in humans. Additionally, the rapid stimulation of whole-body glucose disposal typically occurs in the range of ~2–5 x basal and depends on the amount of ingested glucose [[16], [17], [18], [19],^28^,[36], [37], [38]]. Furthermore, in healthy humans [16,17] and patients with T2D [38], EGP can be rapidly and robustly suppressed, even with low doses (i.e., 25–35 g) of oral glucose, with doses ranging from 25 to 75 g producing near identical degrees of EGP suppression over the first 3 h following ingestion [17]. In contrast, these types of variable infusion ‘dual’ and ‘triple tracer’ postprandial glucose flux experiments are incredibly challenging to perform in mice due to their small body size, limited blood volumes, and extremely high metabolic rate [1]. Thus, it is unknown to what degree EGP is inhibited and glucose disposal is stimulated during a GTT in mice. However, though it is not possible to measure absolute rates of glucose flux by using the siGTT in mice (or rats), it is still possible to gather quantitative and qualitative data on the patterns of EGP and glucose disposal, which can expound on the alterations in glucose flux that occur following glucose administration [[20], [21], [22], [23], [24], [25], [26], [27],[39], [40], [41], [42]].

Here, our aim was to compare siOGTT data from young adults with normal and impaired glucose tolerance (IGT) and impaired fasting glucose (IFG) to siOGTT data from conscious chow and high-fat high-sucrose fed C57Bl/6 mice. The OGTT was chosen as it is the most physiologically and clinically relevant form of the GTT.

## 2 Methods

### 2.1 Human studies

A detailed description of the study cohort is available from a previously published study [15]. Briefly, 254 healthy (BMI<30 kg/m^2^) young adults aged between 18 and 35 years, without a prior diagnosis of prediabetes or diabetes, were studied. Participants were recruited from Deakin University and surrounding areas. The study was approved by the Deakin University Human Research Ethics Committee, and informed written consent was obtained prior to participation. Participants were studied in the morning (08:00–09:00 h) after an overnight (~10 h) fast. A catheter (22 gauge) was inserted into a forearm vein for venous blood sampling. After obtaining baseline blood samples (−10 and 0 min), a 75 g glucose test drink (Daniels Health, Dandenong South, VIC, Australia) enriched with >99% pure [6,6-^2^H_2_]glucose (4% wt/vol; Cambridge Isotope Laboratories, Tewksbury, MA, USA) was consumed. Blood was sampled at 10, 20, 30, 60, 90, 120, 150, and 180 min after glucose ingestion. Blood samples were immediately placed on ice, later spun in a centrifuge, and plasma stored at −80 °C. Plasma glucose was determined using the glucose oxidase method. Plasma insulin was measured by a commercially available ELISA (ALPCO, Salem, NH, USA). Plasma FFAs were determined using an enzymatic, colourimetric assay (NEFA C Kit; Wako Chemicals, Richmond, VA, USA). Plasma and test drink [6,6-^2^H_2_]glucose enrichment was determined via positive chemical ionisation (methane) gas chromatography mass spectrometry (GC–MS) according to previously published methods [17]. Based on the results of the fasting glucose and OGTT analysis, 33 individuals were identified with prediabetes (Table 1), 14 with isolated impaired fasting glucose (IFG; fasting glucose 6.1–7.0 mM), and 19 with isolated impaired glucose tolerance (IGT; 2 h OGTT glucose 7.8–11.1 mM; fasting glucose <6.1 mM).

**Table 1:** Participant characteristics.

	NGT	IFG	IGT
N (F/M)	221 (130/91)	14 (6/8)	19 (15/4)
Age	24.7 ± 0.3	23.5 ± 1.2	24.5 ± 1.1
Body mass (kg)	69.7 ± 13.4	74.9 ± 3.5	67.0 ± 3.3
Height (m)	1.71 ± 0.01	1.76 ± 0.02^†^	1.66 ± 0.02
BMI	23.6 ± 0.2	23.9 ± 0.8	24.1 ± 0.8
Fasting glucose (mM)	5.3 ± 0.1	6.3 ± 0.1∗^†^	5.5 ± 0.1
Fasting insulin (pM)	24.9 ± 2.4	29.1 ± 6.6	41.7 ± 8.3
Fasting FFA (mM)	0.26 ± 0.01	0.22 ± 0.03	0.34 ± 0.04∗

∗P < 0.05 vs. NGT; †P < 0.05 IFG vs IGT. Data are mean ± SEM. Data were analysed by one-way ANOVA followed by Tukey's multiple comparisons test.

### 2.2 Mouse studies

Four-week-old male C57Bl/6 mice (N = 89; Animal Resources Centre, Australia), were maintained at 22 ± 1 °C on a 12/12 h light/dark cycle, with free access to water and a standard chow diet (5% energy from fat, 12.6 kJ/g; Barastoc Rat & Mouse, Ridley AgriProducts, Australia). Mice were housed 4–5 per cage and were acclimatised to the facility for 4 weeks prior to initiating experiments. At 8 weeks of age, mice were randomly allocated to one of two dietary conditions, either maintained on a chow control diet (N = 43) or switched to a high-fat high-sucrose diet (HFHS; N = 46; 42% energy from fat and 20% from sucrose, 19 MJ/kg**;** Specialty Feeds SF4-001, Australia) for 8 weeks. Experiments were approved by the Deakin University Animal Ethics Committee and performed according to the Guide for the Care and Use of Laboratory Animals, Eighth edition (2011).

Body weight was measured weekly and an OGTT performed in conscious mice at the end of the 8-week study. In the two weeks prior to the OGTT, sham gavages were performed on all mice by the same handler at 2- or 3-day intervals in the same procedure room (OGTT room). This procedure involved scruffing mice and performing an oral gavage with an empty syringe. The tails of the mice were also gently ‘milked’ to simulate what would occur during the blood collection period during the OGTT. This routine familiarised the mice with the OGTT procedure and handler in an attempt to minimise the stress response. On the day of the OGTT procedure, following a 5 h fast (food removed at 07:00 h), mice were weighed, and a blood sample (~30 μL) was obtained from the tail vein in the conscious state to measure fasting blood glucose, plasma insulin, and free fatty acids (FFA). A stable isotope labelled OGTT was then performed [22]. Glucose (50 mg of [6,6-^2^H_2_]glucose; dissolved in 200 μL H_2_O; Cambridge Isotope Laboratories, Tewksbury, MA, USA) was administered via oral gavage to conscious mice, and blood was obtained at 15, 30, 45, 60, 90, and 120 min. Blood glucose was measured using a hand-held glucose metre (Accu-Check, Roche, NSW, Australia). Blood (~30 μL) was obtained via a capillary tube to measure plasma insulin and FFAs at 15  and 45 min after glucose administration. Smaller blood samples (~5 μL) were also obtained prior to and at 15, 30, 60, and 120 min after glucose gavage to determine glucose tracer enrichment by positive chemical ionisation (methane) GC–MS as previously described [22]. Plasma insulin was measured by ELISA (Millipore), whereas FFAs were measured spectrophotometrically by an enzymatic colourimetric assay (NEFA C Kit; Wako Chemicals).

A separate group of chow-fed male C57Bl/6 mice (eight weeks old; n = 8) were familiarised for two weeks by the same handler, and the OGTT procedure was performed as described above to determine the acute metabolic response associated with the OGTT procedure and related handling stress. At 10 weeks of age, following a 5 h fast (food removed at 07:00 h), a blood sample (~30 μL) was obtained from the tail vein in the conscious state to measure blood glucose, plasma insulin, and free fatty acids (FFA). Shortly after, mice were scruff restrained and administered 200 μL of H_2_O via oral gavage (sham OGTT), and tail vein blood was again collected at 5 and 15 min after H_2_O administration.

### 2.3 Glucose tracer calculations

Raw GC–MS data were corrected for natural isotopic background abundance skew by analysing unlabelled glucose standards and basal (pre-OGTT) plasma samples via the matrix method [43]. After correcting for natural isotopic abundance in each participant, the measured plasma fractional M+2 enrichment ([6,6-^2^H_2_]glucose) at each time point was divided by the measured fractional M+2 enrichment ([6,6-^2^H_2_]glucose) in the corresponding glucose test drink. The ‘drink corrected’ fractional value was then multiplied by the total blood glucose concentration at each corresponding time point of the OGTT to calculate absolute exogenous glucose concentrations. Endogenous glucose concentrations were calculated by subtracting the exogenous glucose concentration from the total blood glucose concentration. Fractional endogenous glucose enrichment was calculated by dividing the endogenous glucose concentration by the total blood glucose concentration. The same calculations were also performed in the mouse studies, except that plasma M+2 enrichment was not ‘drink corrected.’ Due to their small size and, thus, low tracer cost, it was possible to administer mice with pure (>99%) [6,6-^2^H_2_]glucose.

### 2.4 Statistics

Results are either presented as individual data or mean ± SEM. Data were analysed by an independent t-test, one way ANOVA (followed by Tukey's multiple comparison test), repeated measures one way ANOVA (followed by Tukey's multiple comparison test), or two-way repeated measures ANOVA (followed by Holm-Sidak's multiple comparisons test) where appropriate. The incremental area under the curve (iAUC) for the blood glucose response during the OGTT was determined using the trapezoidal method. Statistical significance was set at P < 0.05.

## 3 Results and discussion

### 3.1 Human OGTT responses

**Figure 1:**
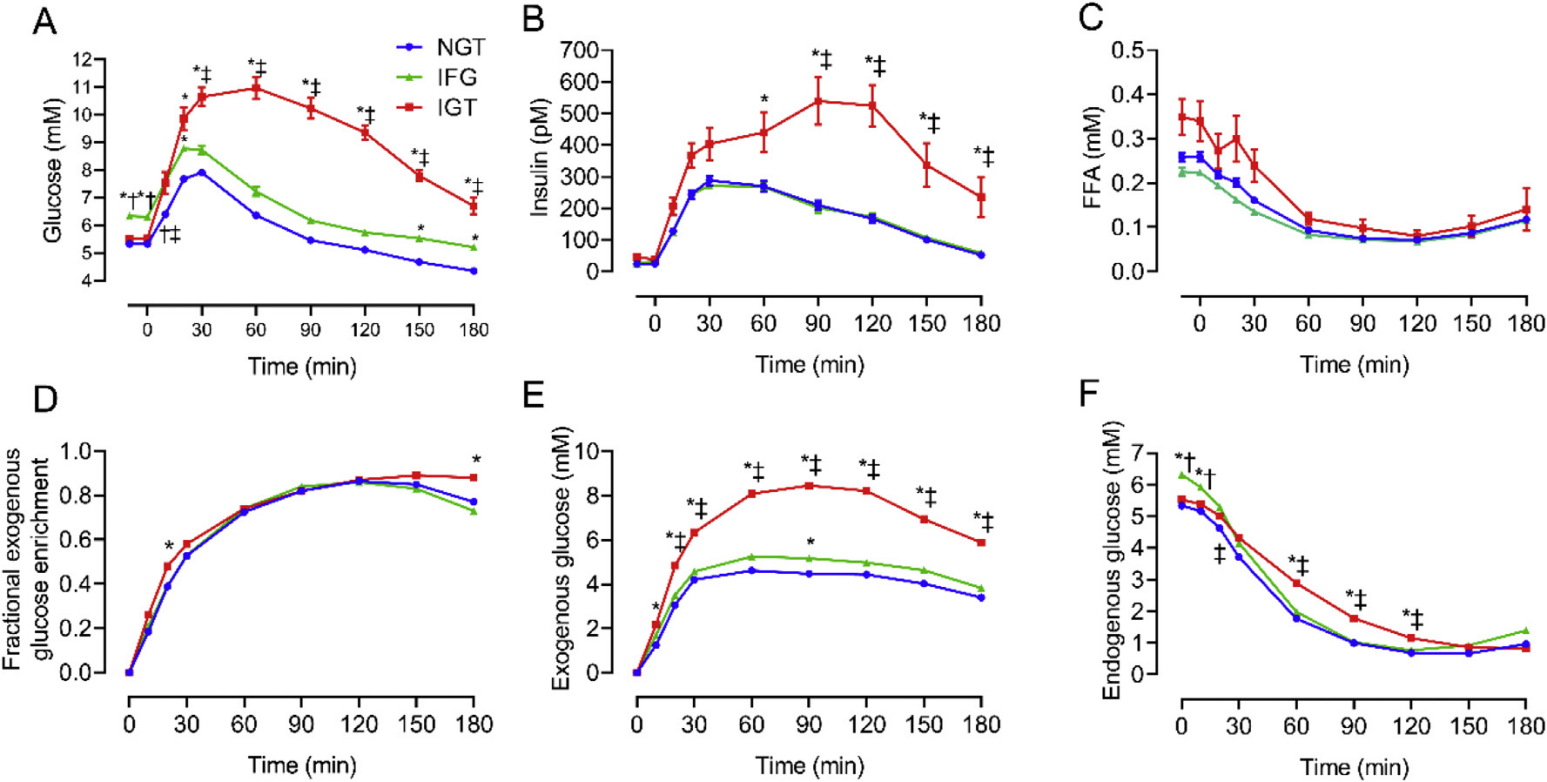
In humans, glucose (A), insulin (B), FFA (C), exogenous glucose enrichment (D), exogenous glucose concentrations (E) and endogenous glucose concentrations (F) during the OGTT in individuals with NGT (n = 221), IFG (n = 14) and IGT (n = 19). Data are mean ± SEM. Data were analysed by two-way repeated measures ANOVA. Significant (P < 0.05) main effects for time and group as well as time × group interactions were found for all data. ∗P < 0.05 vs. NGT; †P < 0.05 vs. IGT; ‡P < 0.05 vs. IFG.

Participant characteristics are presented in Table 1. In accordance with WHO criteria, subjects with normal overnight fasting (<6 mM) and 120 min post-load glucose concentrations (<7.8 mM) are referred to as having normal glucose tolerance (NGT group; Figure 1A). In the NGT group, plasma insulin levels during the siOGTT peaked at ~30 min (11-fold basal; Figure 1B), corresponding to the peak plasma glucose concentration occurrence (Figure 1A). Insulin levels did not return to baseline during the 180 min experimental period, remaining ~7- and 2-fold higher than basal levels at 120 and 180 min, respectively (Figure 1B). As expected (and consistent with robust inhibition of adipose tissue lipolysis), plasma FFA concentrations were significantly reduced during the siOGTT, reaching a nadir between 60 and 90 min and remained in a suppressed state until the end of the experimental period (180 min; Figure 1C).

The NGT group [6,6-^2^H_2_]glucose enrichment data (Figure 1D) demonstrated that within 10 min of glucose ingestion, ~20% of the total blood glucose pool was exogenously derived, corresponding to an exogenous glucose concentration of ~1 mM (Figure 1E). Plasma exogenous glucose enrichment progressively increased beyond this point, peaking at around 120 min, with only a modest decline thereafter (Figure 1E). The endogenous glucose concentration rapidly declined and remained suppressed (Figure 1F). Several important conclusions can be made from the tracer data regarding glucose handling in the NGT group. Firstly, exogenous glucose rapidly replaced the endogenous (EGP derived) plasma glucose pool, indicating a rapid and robust suppression of EGP. Indeed, within 120 min following glucose ingestion, ~90% of plasma glucose was orally derived (Figure 1D), corresponding to an exogenous glucose concentration of ~4.5 mM (Figure 1E), whereas endogenous levels were only ~0.5 mM (Figure 1F). Thus, even at 180 min after glucose ingestion, EGP remained in a suppressed state, and there was continued entry of gut-derived exogenous glucose into the systemic circulation. This finding is consistent with previous human variable infusion ‘dual’ and ‘triple’ tracer experiments. Specifically, we [[17], [18], [19]] and others [36,37,[44], [45], [46], [47]] have shown that at 180 min following glucose ingestion (≥75 g), 1) EGP remains suppressed by 50–75% of the basal pre-ingestion (postabsorptive) values, 2) gut-derived glucose continues to appear in the plasma, and 3) whole-body glucose disposal rates remain substantially elevated above basal.

Concerning participants with IFG (i.e., fasting glucose 6.1–6.9 mM with 120 min glucose <7.8 mM) and isolated IGT (fasting glucose <6.1 mM but 120 min glucose >7.8 mM), it is apparent that the metabolic responses are divergent, consistent with previous literature that demonstrates they are distinct entities [47]. Specifically, compared to the NGT group, the only major metabolic change in subjects with IFG was that fasting glucose concentrations were moderately elevated, also translating to a proportionally elevated glucose excursion during the OGTT (Figure 1A). Apart from this higher ‘glucose set point,’ fasting and post glucose load insulin and FFA levels did not differ from the NGT group (Figure 1B–C), nor did the exogenous enrichment or concentration of exogenous and endogenous glucose (Figure 1D–F). Therefore, based on this data and consistent with previous findings by others [47] and our published modelling for this population [15], subjects with IFG have relatively normal postprandial EGP suppression, gut glucose appearance, glucose disposal, insulin secretion, and insulin action. Together, these findings suggest that the major change in subjects with IFG is a fasting β-cell ‘glucose set point’ abnormality with a normal β-cell insulin secretory response to glucose ingestion [15,47].

Conversely, subjects with isolated IGT displayed more pronounced metabolic changes that differed substantially from the NGT and IFG groups. The isolated IGT group had normal fasting glucose but abnormally high glucose excursions during the siOGTT (Figure 1A), accompanied by hyperinsulinemia under both fasting and OGTT conditions (Figure 1B). Fasting FFA concentrations were modestly elevated in the IGT group, though FFA suppression was normal (Figure 1C). Subjects in the IGT group had a very similar rise in exogenous glucose enrichment as those with NGT, with slight differences only evident at the 20- and 180-min time points (Figure 1D). The decline of the endogenous glucose concentration during the siOGTT was moderately delayed in subjects with IGT, though the same nadir seen in the NGT and IFG group was ultimately achieved within 150 min after glucose ingestion (Figure 1F). Participants with IGT exhibited a marked accumulation of exogenous orally-derived glucose in the blood following the siOGTT, with peak exogenous plasma glucose concentrations being nearly double that of the NGT and IFG groups (Figure 1E).

Interestingly, despite the large difference in the absolute exogenous glucose concentrations of the IGT group (Figure 1E), the proportion of glucose derived from the oral glucose load (i.e., fractional exogenous glucose enrichment) was remarkably similar between the NGT, IFG, and IGT groups, with 70–90% of the blood glucose pool being orally-derived between the 60–180 min time points (Figure 1D). Therefore, based on the observation during the siOGTT of a moderately delayed but ultimately normally suppressed endogenous glucose concentration and a marked elevation in exogenous glucose concentrations, subjects with IGT had relatively normal and robust postprandial EGP suppression but quantitatively significant impairments to postprandial glucose disposal. This finding is consistent with previous studies of IFG and IGT that employed the variable infusion ‘triple tracer’ method, whereby absolute postprandial glucose flux rates were determined [47]. Specifically, subjects with IGT had relatively normal EGP suppression but a transiently-impaired stimulation of glucose disposal immediately following meal ingestion [47]. The early and transient delay in the postprandial stimulation of glucose disposal explains the heightened accumulation of glucose in the blood in IGT, with subsequent hyperglycemia normalising (i.e., compensation via glucose effectiveness) rates of glucose disposal in the later postprandial period [1,47]. However, it should be highlighted that in our IGT group, the combined existence of fasting and postprandial hyperinsulinemia, as well as postprandial hyperglycemia, suggests the presence of both hepatic and peripheral tissue insulin resistance and inappropriate β-cell function relative to the degree of insulin resistance. This finding explains our previously published observations of a markedly reduced disposition index in subjects with IGT [15].

### 3.2 Mouse OGTT responses

siOGTT's were performed on a large cohort of chow and HFHS-diet-fed male C57Bl/6 mice to gain insight into glucose handling during the OGTT in the most used mouse model of metabolic disease. Prior to the dietary intervention, both groups had identical body mass (Table 2). At the end of the 8-week dietary intervention, the HFHS-diet-fed mice gained significantly more body weight than the chow controls (Table 2). All animals were acclimatised to handling to try minimise the stress associated with performing the OGTT, as handling stress can have negative impacts on the outcomes of metabolic tests in mice [[7], [8], [9], [10], [11], [12]]. Accordingly, two weeks prior to undergoing the siOGTT, mice were handled (i.e., scruffed and sham gavaged) by the same handler every 2–3 days. A fixed dose of glucose (50 mg) was administered to mice, consistent with the fixed dose (i.e., 75 g) clinical OGTT standards for humans. The 50 mg fixed GTT dose has been recommended by numerous groups [7,10] and has proven useful for phenotyping mice in our studies [[20], [21], [22], [23], [24], [25], [26], [27]]. Importantly, the fixed dose GTT avoids the bias (i.e., ‘glucose over dosing’) introduced when studying obese mice that have greater total body mass yet similar lean mass to their lean chow-fed counterparts compared to dosing relative to total body mass (i.e., grams glucose/kg body mass; [[7], [8], [9], [10], [11], [12]]). Based on our previously published body composition (EcoMRI) data [[48], [49], [50], [51], [52], [53]], adult male C57Bl/6 chow- and HFHS-fed mice typically have ~25 g of lean body mass (LBM); thus, the 50 mg fixed dose approximates to 2 g/kg/LBM. Dosing relative to LBM is also useful for minimising the bias associated with assessing glucose tolerance in obese mice [7,11,12]. Notably, we have found that the 50 mg and 2 g/kg/LBM OGTTs yield near identical glucose excursions [23].

**Table 2:** Metabolic characteristics of chow and HFHS fed mice.

	Chow (n = 43)	HFHS (n = 46)
Starting body mass (g)	24.3 ± 0.3	24.3 ± 0.3
Final body mass (g)	30.7 ± 0.5	40.1 ± 0.9∗
Change in body mass (g)	6.3 ± 0.3	15.7 ± 0.7∗
Fasting glucose (mM)	8.0 ± 0.2	10.2 ± 0.2∗
Fasting insulin (pM)	330 ± 33	608 ± 72∗
Fasting FFA (mM)	1.13 ± 0.03	0.82 ± 0.03∗

∗P < 0.001 vs Chow. Data are mean ± SEM. Data were analysed by an independent t-test.

**Figure 2:**
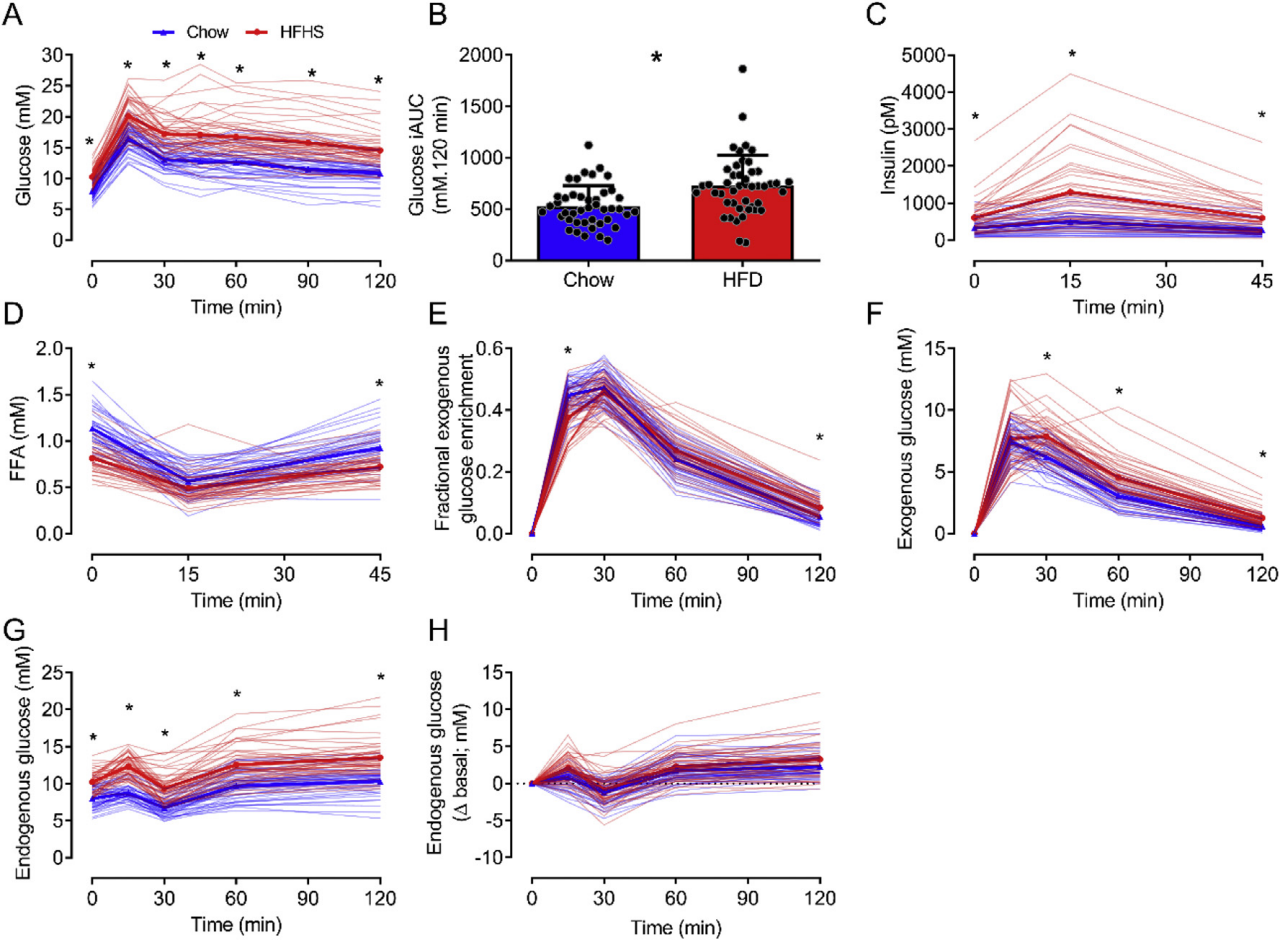
In mice, glucose (A), glucose iAUC (B), insulin (C), FFA (D), exogenous glucose enrichment (E), exogenous glucose concentrations (F), endogenous glucose concentrations (G) and the change in endogenous glucose concentration from basal (H) during the OGTT in chow (n = 43) and HFHS (n = 46) fed conditions. Individual data (thin blue and red lines) and the mean (thick blue and red lines) are presented in A and C–I. Individual data and mean ± SEM are presented in B. Data in A and C–I were analysed by two-way repeated measures ANOVA. Significant (P < 0.05) main effects for time and group as well as time × group interactions were found for all data. Data in B were analysed by an independent t-test. ∗P < 0.001 vs HFHS.

Compared to chow, the HFHS diet caused both fasting and postprandial hyperglycemia (i.e., increased glucose iAUC) and hyperinsulinemia (Table 2 and Figure 2A–C). Despite being obese, hyperglycemic, and hyperinsulinemic, fasting plasma FFAs were lower in HFHS-fed mice (Figure 2D). However, during the siOGTT, FFA levels were transiently suppressed to the same degree in both groups (Figure 2D). This outcome was consistent with our previous findings [[21], [22], [23],^52^] and those in other mouse [54] and rat studies [55,56], demonstrating that HFHS feeding lowers fasting FFAs.

During the siOGTT, fractional exogenous glucose enrichment rapidly peaked between 15 and 30 min in both the chow- and HFHS-fed mice (Figure 2E), After this time, fractional exogenous glucose enrichment rapidly declined, and by the end of the siOGTT (120 min), enrichments in both groups were <10% (Figure 2E). The fractional exogenous glucose enrichment excursions were overall similar across both diets, with only modest differences at 15 and 120 min. The overall pattern of the endogenous glucose excursion was similar for both diets, albeit absolute concentrations were higher in HFHS-fed mice, which appears to be related to their fasting hyperglycemia (Figure 2G). This effect is illustrated by examining the change in endogenous glucose concentration from baseline (Figure 2H), in which the responses for the chow and HFHS groups were similar. The HFHS-fed mice also exhibited an accumulation of orally-derived exogenous glucose in the blood compared to chow controls (Figure 2F), consistent with reduced stimulation of postprandial glucose disposal.

### 3.3 Species comparison

When comparing the mouse and human siOGTT responses, it is apparent there are some major species differences. Firstly, in humans, glucose ingestion stimulates a large (≥11-fold basal at peak) and sustained (above basal ≥ 150 min) rise in insulin, eliciting a robust and persistent suppression of lipolysis, as evident by the drop in plasma FFAs (>50% suppression for >180 min). In contrast, the magnitude of the insulin response (≤2-fold basal at peak) and suppression of FFAs (≤50%) in mice during the OGTT was somewhat small. Furthermore, both responses were transient, with plasma insulin and FFAs returning to baseline levels within 45 min of glucose administration. Significantly, humans and mice also exhibit different glucose handling mechanisms following glucose administration, which will be discussed in detail below.

Unlike humans, in mice, the orally-derived glucose is rapidly absorbed and cleared from the blood. This effect is apparent when comparing the mouse and human exogenous glucose enrichment excursions (Figure 1D vs Figure 2E). In humans, peak enrichments occurred at ~120 min after glucose ingestion and remained at this level for another hour. However, in mice, peak enrichments occurred within ~30 min of glucose administration and rapidly declined. Moreover, in humans, at 120 min after glucose administration, ~90% of the blood glucose pool was orally derived, whereas at this same time point in mice, <10% was of oral origin. The rapid clearance of orally-derived glucose in mice is consistent with mice possessing basal metabolic and glucose turnover rates ~7–10 times (in relative terms) higher than humans [1].

It is important to highlight that a fundamental principle of tracer dilution methodology is that glucose enrichment is only influenced by glucose appearance and not glucose uptake, as cells do not discriminate between labelled and unlabelled glucose during the uptake process [57]. Therefore, a drop in plasma glucose enrichment can only occur if labelled glucose is diluted by the appearance of unlabelled endogenous glucose into the bloodstream. Thus, when interpreting the glucose enrichment data, significant species differences exist regarding the regulation of EGP during an OGTT. Indeed, the persistently high enrichment levels in humans demonstrate robust and sustained EGP suppression during the OGTT. In contrast, the rapid dilution of glucose enrichment in mice illustrates that EGP still occurs at high rates during an OGTT.

**Figure 3:**
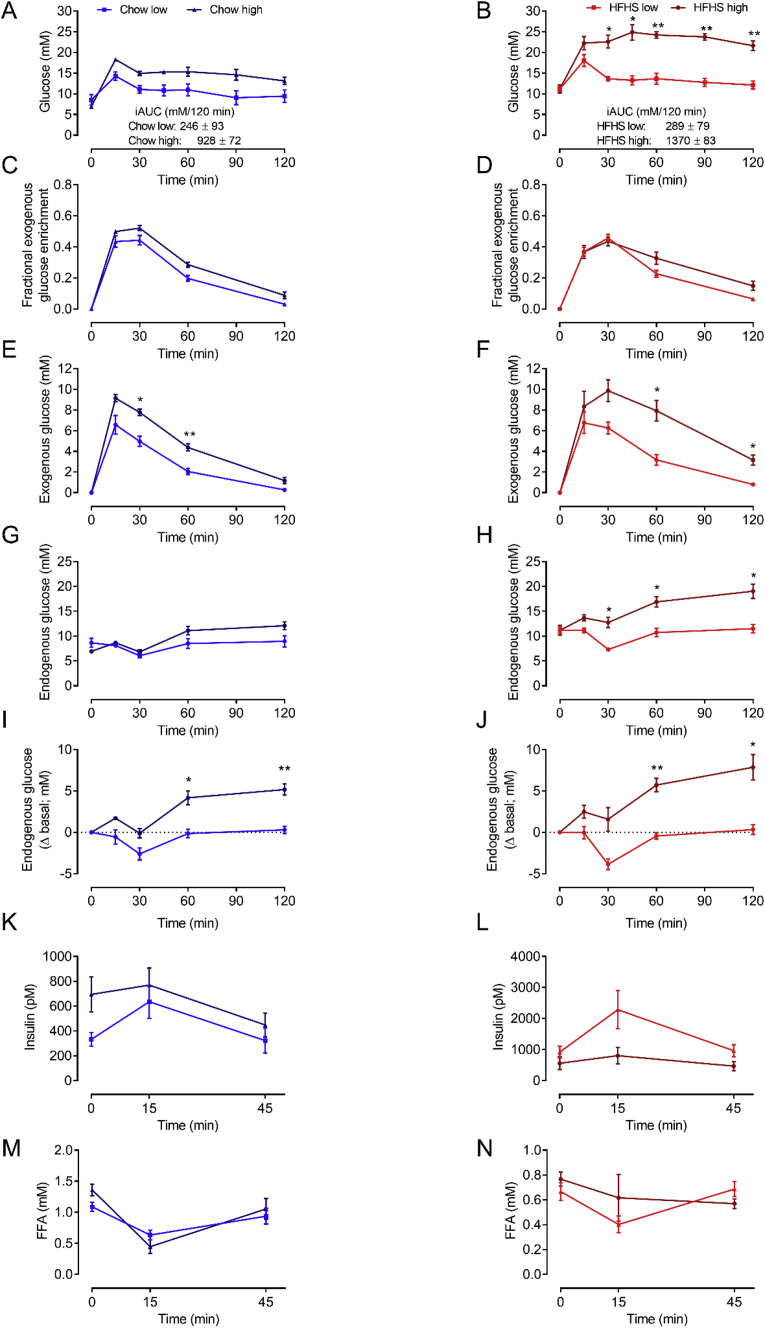
In mice, glucose (A & B), exogenous glucose enrichment (C & D), exogenous glucose concentrations (E & F), endogenous glucose concentrations (G & H), change in endogenous glucose concentration from basal (I & J), insulin (K & L) and FFA (M & N) during the OGTT in chow (A, C, E, G, I, K, M) and HFHS (B, D, F, H, J, L, N) fed conditions with the lowest and highest glucose iAUC. N = 4 for each group. Data are mean ± SEM. Data were analysed by two-way repeated measures ANOVA. Significant (P < 0.05) main effects for time and group as well as time × group interactions were found for data in A, B, E, F, and H. Significant (P < 0.05) main effects for time and time × group interactions were found for data in G and L. Significant (P < 0.05) main effects for time and group were found for data in C. Significant (P < 0.05) main effects for time were found for data in D, K, and M. ∗P < 0.05 vs low; ∗∗P < 0.01 vs low.

The key differences in human and mouse EGP regulation during the OGTT are also evident by comparing the endogenous glucose concentration excursions (Figure 1F vs Figure 2G). In humans, all study participants (i.e., irrespective of NGT, IFG and IGT) exhibited robust and sustained reductions in endogenous glucose concentrations, and endogenous glucose levels remained suppressed at the end of the siOGTT (i.e., 180 min; Figure 1F). In contrast, endogenous glucose concentrations in mice, regardless of diet, were, on average, minimally and transiently suppressed (Figure 2G,H). Notably, when examining individual mouse responses, ~26% of chow- and ~35% of HFHS-fed mice showed no reduction in endogenous glucose concentrations. Interestingly, endogenous glucose concentrations persistently increased during the OGTT in some mice, demonstrating a paradoxical increase in EGP (not just lack of EGP suppression). This effect prompted us to further examine mice at either end of the glycemic control spectrum to ascertain whether this paradoxical increase in endogenous glucose concentration is a characteristic feature of mice with the worst glucose tolerance. To do this, mice in both the chow and HFHS groups were stratified according to their blood glucose excursion during the OGTT (i.e., <10th centile glucose iAUC ‘low excursion’ vs > 90th centile glucose iAUC ‘high excursion’; Figure 3). Irrespective of diet, this analysis revealed that mice with the highest glucose excursions (i.e., worst glucose tolerance) had paradoxically rising endogenous glucose excursions, which was most pronounced on the HFHS diet (Figure 3G–J). Similarly, the most glucose-intolerant mice had the greatest exogenous glucose excursions, which was again most pronounced in the HFHS group (Figure 3E,F). This result indicates that the manifestation of severe glucose intolerance in male C57Bl/6 mice is not only driven by the impaired stimulation of tissue glucose disposal but also by a paradoxical increase in EGP, which is markedly different to what occurs in glucose-intolerant humans. Interestingly, irrespective of diet, neither ‘low’ nor ‘high’ mouse groups had statistically significant differences in body mass (chow ‘low’ 27.5 ± 1.7 g; chow ‘high’ 28.5 ± 1.0 g; HFHS ‘low’ 41.3 ± 4.3 g; HFHS ‘high’ 36.8 ± 1.9 g), insulin (Figure 2K & L), or FFA (Figure 2M & N).

### 3.4 So, how can these marked species differences in postprandial metabolism during the OGTT be explained?

**Figure 4:**
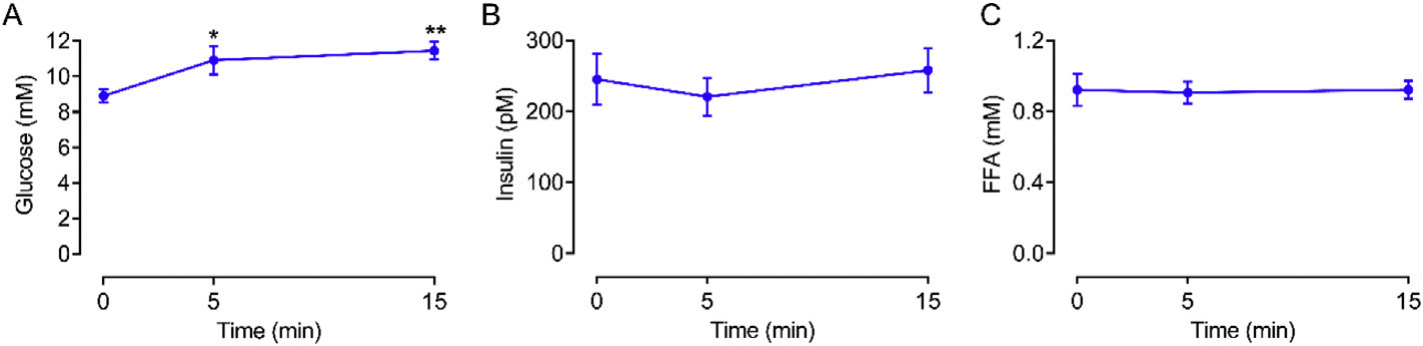
In mice, glucose (A), insulin (B) and FFA (C) concentrations during a sham water gavage (n = 8). Data are mean ± SEM. Data were analysed by one-way repeated measures ANOVA. The ANOVA for the glucose data in (A) was statistically significant (P = 0.02). ∗P < 0.05 vs. 0 min; ∗∗P < 0.0001 vs. 0 min.

This finding is most likely explained by understanding that mice are small prey animals, making them highly vulnerable to handling stress [[7], [8], [9], [10], [11],^14^]. Stress stimulates catecholamine secretion and this impacts glucose metabolism by increasing glycogenolysis in liver and skeletal muscle which stimulates hepatic glucose production while also inhibiting blood glucose uptake in the muscle and liver [58]. Catecholamines also potently inhibit insulin secretion and stimulate lipolysis [58]. In addition, corticosterone secretion increases with handling stress in mice, which is relevant considering that corticosterone can stimulate EGP and inhibit glucose uptake and insulin secretion [59]. Collectively, stress can promote hyperglycemia [58,59]. Unfortunately, we did not measure plasma catecholamines or corticosterone due to inadequate sample volume following the plasma glucose tracer, insulin, and FFA analysis. However, endogenous glucose concentrations either dropped very little (~26% chow and ~35% HFHS mice did not drop at all) or, in some mice, markedly increased (~10% of mice; both diets), suggesting the mice experienced a significant degree of handling stress. Hence, despite familiarisation with the OGTT procedure, combined with our experience in handling and phenotyping of mice, it appears we were not able to mitigate the stress response. Interestingly, the lack of a reduction in endogenous glucose concentrations during the siOGTT in mice also appears to occur following intraperitoneal glucose administration [40]. This finding suggests the stress response was not due to the gavage or injection procedures but due to handling (i.e., scruffing and tail blood sampling; [[7], [8], [9], [14]]). Furthermore, C57BL/6 mice appear to be particularly sensitive to stress-induced hyperglycemia [60,61]. Indeed, the pioneering work of Surwit and colleagues showed that C57BL/6 have heightened hyperglycemic responses to stress and catecholamine administration, and these responses are exacerbated by obesity [60,61]. Other more recent studies have also shown that obese C57BL/6 also have exaggerated corticosterone and hyperglycemic responses upon restraint [59]. This inherent stress response can be seen in our own experiments (Figure 4), in which chow-fed male C57BL/6 mice that were familiarised with handling procedures underwent an oral gavage to administer water (i.e., no glucose sham treatment). Following the gavage of water, blood glucose rapidly increased, whereas insulin and FFAs were unaffected (Figure 4). Thus, it appears that C57BL/6 mice are sensitive to handling (e.g., restraint, gavage, injection, tail sampling) stress-induced hyperglycemia.

It is interesting to note that despite a very small and transient (≤2-fold; <45 min) insulin response (Figure 2C), mice effectively clear orally administered glucose (Figure 2A). We believe this finding can be explained by the fact that the disposal of a *glucose bolus* in mice (and rats) occurrs predominantly via an insulin*-independent* process [[62], [63], [64]]. Specifically, following the induction of hyperglycaemia by glucose bolus administration, in the presence of basal insulin levels, the mass action of hyperglycemia (i.e., glucose effectiveness) provides the predominant stimulus for the increase in whole-body glucose uptake, thus facilitating the clearance of exogenous glucose [[62], [63], [64]]. This high level of ‘glucose effectiveness’ likely results from the inherently high metabolic rate that small mammals require to preserve body temperature [1], which means mice can rapidly dispose of an exogenous glucose bolus and normalise glycemia independent of any appreciable rise in insulin secretion (above baseline) and without significant (if any) EGP suppression. This suggests that, in rodents, insulin secretion does not need to undergo large fluctuations between the postabsorptive and postprandial periods, unlike what occurs in humans.

Another factor that may contribute to the efficient disposal of exogenous glucose in mice could be related to stress-induced catecholamine secretion diverting glucose uptake from one tissue to another. Though the handling stress-induced catecholamine response would be expected to prevent EGP suppression (or cause it to rise) and inhibit muscle and hepatic glucose uptake, it could simultaneously increase the metabolic rate and increase glucose uptake into both brown adipose tissue (BAT) and the heart [[65], [66], [67]]. Unlike humans, mice have large, well-defined BAT depots [1], and as catecholamines stimulate BAT glucose uptake in mice [[65], [66], [67]], BAT could be a quantitatively significant site of increased glucose disposal in stressed mice during a GTT. Catecholamines also stimulate cardiac glucose uptake in mice, also potentially making this a site of increased glucose uptake during a GTT in mice experiencing handling stress [65,66,68].

In the context of metabolic experiments, humans are aware of their laboratory surroundings and can remain relaxed during postprandial catheter-based blood sampling studies, evident from our experiments (e.g., [69]) and those of others (e.g., [70]), which show that highly stable euglycemia (i.e., no hyperglycemic response) is maintained during water ingestion control experiments. However, it is difficult to avoid the confounding metabolic effects of stress that occur during the handling of conscious mice during a GTT. Given that C57Bl/6 mice, especially those in an obese state, have heightened stress and catecholamine-induced hyperglycemic responses [60,61], we believe deconvoluting the mechanistic factor(s) that impair or improve glucose tolerance in mice is exceptionally challenging. The fact that conscious handled mice have high metabolic rates, are easily stressed and rely heavily on insulin independent mechanisms to clear exogenous glucose, means GTT responses are fundamentally different from that in humans. In this regard, it is difficult to decipher whether the diet-induced model of prediabetes is more representative of IFG, IGT, or a combination of both and whether it is the loss of insulin action, β-cell function, impaired glucose effectiveness, or handling stress that drives the apparent hyperglycemic state. Similarly, unlike in humans in which there are clear (regardless of age or ethnicity) diagnostic blood glucose and HbA1c criteria defining what constitutes IFG, IGT, and overt diabetes (i.e., WHO or ADA criteria), stringent guidelines constituting normal/abnormal glycemia in mice do not exist.

Though technically challenging, using mouse surgical catheterisation approaches makes it possible to perform metabolic testing in mice with minimal handling, thus avoiding handling-induced catecholamine and/or corticosterone stress responses [8,71]. While these techniques are typically employed during mouse glucose-clamp studies, we are aware of only one report in which catheters were surgically implanted into the stomach (glucose administration) and carotid artery (blood sampling) to enable OGTTs to be performed in conscious unhandled mice [72]. With this approach, fasting glucose and OGTT glycemic excursions were lower, whereas the insulin response was considerably higher [72] than the responses in our study and those typically reported in the literature for conscious handled mice. However, the insulin response was still transient [72], which is very different to humans. It is also noteworthy to highlight that though a specific gene knockout strain of mice used in this study exhibited severe hepatic insulin resistance, as determined by the euglycemic-hyperinsulinemic clamp, this effect did not translate to hyperinsulinemia or hyperglycemia during the GTT [72], supporting the concept that mice are not heavily reliant on the glucose-lowering actions of insulin during a GTT. Though this approach is an effective way to minimise handling stress during the GTT, the catheter implantation surgeries are complex, require specialised staff, and are time consuming. Therefore, this approach is not feasible for most laboratories.

It is essential to highlight some of the study limitations. In interpreting OGTT data, the time of day could be an important consideration. Though our studies in humans were conducted in the morning after an overnight fast, the mice were studied in the middle of their light cycle, which corresponds to experiments being conducted in the middle of their sleep cycle, as mice are nocturnal [13]. Though this method is convenient for researchers and standard practice for the field [8], it could be an additional stressor to the mice. Thus we cannot rule out the impact of the diurnal cycle on glucoregulation. Furthermore, though our human cohort consisted of both male and female participants, our mouse studies only used male mice. As female mice are generally less susceptible to diet-induced glucose intolerance [13], they may exhibit some alterations to glucose handling kinetics compared to male mice, warranting the inclusion of both sexes in future studies.

## 4 Summary and conclusions

Using a siOGTT, we have provided evidence that mice and humans use distinct mechanisms to coordinate the clearance of orally-derived glucose from the circulation. Though mice rapidly clear glucose in the absence of large insulin responses, humans exhibit an extended postprandial period characterised by robust and sustained insulin responses. Furthermore, as EGP is resistant to suppression during an OGTT in mice, they appear to be highly reliant on glucose disposal for the clearance of a glucose load. In contrast, EGP is readily and persistently suppressed in humans in response to oral glucose ingestion. These species differences likely occur due to mice having a small body size, high metabolic rates and that glucose metabolism in C57Bl/6 mice is easily influenced by handling and procedural stress. These findings have important implications for interpreting mouse glucose tolerance test results and translating these findings to humans.

## Author contributions

Conceptualisation: G.M.K. and C.R.B.; Methodology: G.M.K., C.R.B., S.H., K.F.H., C.S.S., and T.A; Investigation: G.M.K., S.H., C.R.B., C.S.S., T.A., and K.F.H.; Writing – Original Draft: G.M.K. and C.R.B, Writing – Review and Editing: G.M.K., C.R.B., S.H., K.F.H., C.S.S., and T.A; Funding Acquisition: G.M.K and C.R.B.

## Data statement

The data that support the findings of this study are available from the corresponding author upon reasonable request.
